# Personality traits, self-efficacy, and friendship establishment: Group characteristics and network clustering of college students’ friendships

**DOI:** 10.3389/fpsyg.2022.916938

**Published:** 2022-09-02

**Authors:** Dongdong Yan, Xi Yang, Huanzhe Zhang

**Affiliations:** ^1^Department of Sociology, School of Ethnology and Sociology, Inner Mongolia University, Hohhot, China; ^2^Institute of Population, School of Economics, Hebei University, Baoding, China; ^3^Department of Sociology and Psychology, School of Public Administration, Sichuan University, Chengdu, China

**Keywords:** personality traits, interpersonal self-efficacy, friendship network, network clustering, ERGM

## Abstract

Friendship establishment was analyzed using constructs from social cognitive theory (self-efficacy and personality traits) and social network theory (reciprocity and triad closure). In further studies, we investigated the effect of personality traits, interpersonal self-efficacy, and network structure on the establishment of friendships. In this study, we used social network analysis method and exponential random graph model (ERGM). The following findings are reported. First, the friendship network of college students had small group characteristics, and the formation of this small group was more based on personality complementarity than similarity. The homogeneity hypothesis of personality was not tenable. Secondly, individuals with dominance or influence personality traits and high interpersonal self-efficacy were more likely to be in the center of the friendship network. Furthermore, personality traits and interpersonal self-efficacy may have interactive effects on the formation of friendship networks. Popularity and activity effects existed in friendship networks, but the reciprocal relationship based on personality traits was not verified. The balance structure can easily explain the agglomeration of friendships in a small range, indicating that small groups of friendships prefer a two-way circular close relationship. Finally, the formation of a friendship network includes the comprehensive process of individual characteristics and endogenous tie formation, which helps us to understand the social population structure and its process over a wider range.

## Introduction

“No man is an island.” Every person in a society should connect with one another. These connections can take many forms, such as friendship, romance, financial relations, or kinship, to build social networks that are the result of the interaction between individual communication, social change, and individual events (Degenne and Lebeaux, 2005). In each phase of life, people develop different types of social networks, and those networks evolve through time. In a young person’s life, school may be the most homogeneous and stable environment for establishing friendship networks, which may last for the rest of their life. The interpersonal social network formed during college may have an impact on students’ academic performance and may even become a source of support in life or the workplace. For example, distant interpersonal relationships have been identified as one of the causes of learning deficiencies (Huang and Lin, 2011). Similarly, if an individual can develop a close peer relationship and a positive social network, it will benefit their learning and identity (Flynn et al., 2016). Given the value of close relationships and friendships, especially the social networks that are formed during college, the present study focused on the unique group of traditional full-time college students and analyzed the network clustering and group characteristics of their friendship relationships and how the friendship network is formed.

What causes the aggregation of the social group structure in the network? Traditional structuralists believe that network structure is the main determinant of human interaction and deny the influence of individual characteristics (Mayhew, 1980). Kilduff and Tsai (2003) further emphasized the role of network structure in creating or hindering social interaction and access to resources. However, these views ignore the fact that human beings are active subjects, and their social relations and environment are affected by their motivation, behavior, and personality (Selden and Goodie, 2018). Not everyone agrees with the strong anti-individualist position of traditional structuralists. Sociologists have long noticed that population aggregation tends to be related to sociodemographic attributes (McPherson et al., 2001). In the process of studying the formation of networks, some sociologists try to observe personal motivation and the tendency to evaluate how these characteristics help shape the network structure (Kadushin, 2002). They believe that attributes and behaviors will affect the choice of network partners, thus affecting the network structure and network relations. Therefore, an increasing number of scholars have begun combining the differences in individual social attributes (Clifton, 2014; Lamkin et al., 2014) with the factors of network structure (Kalish, 2008; Flynn et al., 2010; Selfhout et al., 2010; Emery, 2012) to explain the reasons for the formation of a specific network.

The formation of a friendship network is a selection process that is limited and affected by individual social attributes (such as personality traits), selective mixing (including assortative mixing and disassortative mixing), and network structure of triad closure; for example, friends of friends are more likely to become friends (Goodreau et al., 2009). Among them, individual social attributes mainly include personality differences and self-efficacy (Mischel, 1973; Yang and Lee, 2016), while the network structure mainly involves reciprocity, transitive triples, and cyclic triples (Heidler et al., 2014).

## Theoretical background

### Personality traits and friendship networks

According to social cognitive theory, the two core individual characteristic variables are personality traits and subjective values (Mischel, 1973). The influence of personality traits on the formation of friendship networks seems to have reached a consensus (Selden and Goodie, 2018). A stable personality will not only affect the acquisition of communication skills, such that different individuals show differences in social and friendship networks (Hullman et al., 2010), but may also have a potential impact on other structural attributes in the network, such as centrality, homogeneity, and betweenness, or the formation of more complex structures, such as reciprocity and transitive triadic closure (Goodreau et al., 2009). Research on the influence of Five Factor Model personality traits^1^ on the formation and structure of friendship networks is the most common (Baams et al., 2015; Liou and Hsieh, 2020). The influence of personality traits on the friendship network is reflected in the network scale and the individual’s position in the friendship network.

Personality traits will affect the size and homogeneity of friendship networks. Extraverts or those with a social interaction personality type tend to have better social skills and feel less socially anxious than introverts (Smółka and Szulawski, 2011). Therefore, they will have a wider and more intensive friendship network. Selfhout et al. (2010) found in a longitudinal study that personality similarity had a significant impact on the formation of friendships within 4 months. Likewise, the more similar the extrovert personality of different individuals, the more likely they were to become friends (Feiler and Kleinbaum, 2015), which demonstrates the homogeneity effect of extraversion-oriented friendships. Neuroticism or the corrector personality type tend to be socially anxious and lack social skills, so their friendship networks are smaller and contain fewer connections in the network structure (Smółka and Szulawski, 2011). However, there is also research showing that neuroticism was unrelated to the scale of the network (Totterdell et al., 2008; Zhu et al., 2013). Nevertheless, a consistent conclusion has been that neuroticism has no substantial relationship with homogeneity in making friends (Selfhout et al., 2010; Baams et al., 2015). Agreeable and supporter personality types can help assuage difficult or frustrating social relationships, which will affect the network scale and composition of personal networks (Graziano et al., 1996). Agreeable individuals tend to have a larger network of non-relatives than individuals with less agreeableness. On the homogeneity of making friends, for college students, highly agreeable individuals tended to become friends with other agreeable individuals (homophily; Selfhout et al., 2010); however, for adolescent students, agreeable individuals tended to stay friends with disagreeable others (heterophily; Baams et al., 2015). Openness tends to be the most controversial personality trait of the Big Five personality traits (Digman, 1990). Unlike other traits, openness to experience does not have a strong theoretical and empirical body of literature explaining its effect on social relationships (Demır and Weitekamp, 2007). However, studies have found that openness is correlated with the number of new contacts in the network gained since starting college (Wagner et al., 2014). Furthermore, Selfhout et al. (2010) found that openness had a significant homogeneity effect on friendship. It is possible that conscientiousness could have an effect on social network relations, but the effects are likely not as strong or reliable as those of other personality traits. Conscientiousness neither predicted the network scale of college students’ friendship networks (Totterdell et al., 2008; Zhu et al., 2013) nor showed a relationship with centrality and homogeneity (Selfhout et al., 2010).

The structural effect of personality traits on the formation of a friendship network and the influence of personality traits on the formation of individual friendship networks are also reflected in the network structure. Research showed that extraverts or the social interaction personality type not only nominated more people to become friends, but also were nominated as friends more often. Extroverts tend to form more connections than introverts and occupy more central positions in friendship networks (Zell et al., 2014). Although neuroticism and the corrector personality type affect how individuals perceive their personal network structure, it does not affect the network scale or their position in the structure of these networks (Selfhout et al., 2010; Baams et al., 2015). Agreeable people were found to be more likely to be chosen as friends by others (high in-degree), but there was no effect of agreeableness on nominating others as friends (low out-degree; Selfhout et al., 2010). In the friendship networks of adolescents, agreeableness was not related to in-degree or out-degree (degree centrality means the number of links connected to a node; Baams et al., 2015), and openness has not been shown to be significantly related to betweenness centrality in the friendship network of adolescents or college students (Selfhout et al., 2010; Baams et al., 2015). However, in the four advice/support networks of teams in a manufacturing organization, openness to experience correlated with in-degree centrality (Neubert and Taggar, 2004). In friendship networks, the relationship between conscientiousness and high-order network structure has rarely been studied. However, utilizing diverse samples across many types of organizations and situations, it was found that conscientiousness was unrelated to the degree of centrality in advice and support networks in the workplace (Neubert and Taggar, 2004; Daly et al., 2014).

Personality traits play a variety of roles in the formation of friendship networks, which can be regarded as a remote predictor of network perception and structure (Totterdell et al., 2008; Liu and Ipe, 2010). From this theoretical background, we enunciate the three following hypotheses that concern the links between scale, position of friendship networks and personality traits:

**Hpy.1.** Different personality traits would have a significant impact on the scale of the friendship network.

**Hpy.2.** Individuals with similar personality traits would be more likely to become friends (homogeneity).

**Hpy.3.** Extraverts would be more likely to be in the center of the friendship network.

### Self-efficacy and friendship networks

The formation of a friendship network is not only affected by personality traits; self-efficacy is also an important factor (Locke and Sadler, 2007; Hullman et al., 2010). Self-efficacy is a person’s belief that they can use a certain skill, rather than a direct measure of the skill itself (Bandura, 1977). Self-efficacy affects people’s thoughts, self-esteem, goal setting, how much energy they spend, and their choices (Bandura, 1989). Self-efficacy has been widely researched in many areas of human functioning, such as interpersonal communication, learning, working, and love. Moe and Zeiss (1982) developed a scale to assess interpersonal self-efficacy as early as 1982. This concept and scale have also been widely used (Hullman et al., 2010). Interpersonal self-efficacy is a subjective evaluation of an individual’s ability to communicate with others, which affects the individual’s perception of their competence in their communication ability, anxiety in the social process, and the interpersonal relationship (Sandler et al., 2000). Scholars have found a significant positive correlation between social skills and interpersonal self-efficacy (Segrin and Taylor, 2007).

Interpersonal self-efficacy not only affects the scale of friendship networks, but also affects the position of individuals in such networks (Locke and Sadler, 2007). Regarding the scale effect of interpersonal self-efficacy on the formation of friendship networks, Krämer and Winter (2008) showed that self-efficacy had a significant impact on the number of friends, such that the latter increased with an increase in former. People with high interpersonal self-efficacy can deal better with obstacles; it is easier for them to use a variety of ways to deal with or avoid social-related stress and negative emotions, which helps them develop a larger scale of social networks (Snyder, 2002). Studies have shown that individuals with a high level of interpersonal self-efficacy are expected not only to engage in positive behaviors in a new social environment, such as initial contact and participation in group activities (Smith and Betz, 2000), but also to participate in prosocial behaviors, such as helping others, being kind to others, and cooperative behaviors (Bandura et al., 1999). Therefore, individuals who have high interpersonal self-efficacy are more likely to develop larger social networks than those who lack interpersonal self-efficacy. However, the increase of social isolation caused by the COVID-19 has increased people’s social anxiety and significantly reduced the interpersonal self-efficacy, which hand led to the decline in the number of friendship relationships the scale of friendship networks (Smith et al., 2022).

Another consideration is the structural effect of self-efficacy on the formation of friendship networks. People with high interpersonal self-efficacy can adapt better to their environment. At the same time, the process of peer influence suggests that individuals connected to peers with high levels of self-efficacy tend to develop higher efficacy beliefs as well (Siciliano, 2016), which makes them more likely to be in the center of social networks. Moreover, self-efficacy affects individuals’ perceived social support, and a significant positive correlation has been found between perceived social support and social skills evaluated by peers (Sarason et al., 1986). Therefore, individuals with higher self-efficacy will have closer social interaction relationships and may obtain more benefits in return (e.g., social support) in the process of making friends to create and maintain supportive interpersonal relationships (Bandura et al., 1999). Conversely, a larger social network has the potential to provide more social support for individuals with high self-efficacy when needed, making them the center of the network (Zhao et al., 2010). However, the social support was weakened during the COVID-19 pandemic (Elmer et al., 2020). As a result, the central position of individual friendship network will be affected (Diamanti et al., 2021).

It is likely that individuals with high interpersonal self-efficacy would have less fear associated with the process of social communication (Hullman et al., 2010). Consequently, self-efficacy can produce transmission effects, bring high social support and prosocial interaction, and finally produce positive interpersonal communication. From this theoretical background, we issue the following two hypotheses that concern the links between scale, position of friendship networks and interpersonal self-efficacy:

**Hpy.4.** Individuals with higher interpersonal self-efficacy would be associated with a larger scale friendship network.

**Hpy.5.** Individuals with higher interpersonal self-efficacy would be more likely to be in the center of the friendship network.

### Network structure and friendship networks

An increasing number of scholars have begun to break through traditional structuralism and bring individual social attributes and other factors into the process of network analysis. With the development of social network analysis methods and technologies, the impact of network structure (endogenous processes) on network formation is also considered. One of the core findings of social network research is that individual beliefs and behaviors do not result from personal attributes alone (Kilduff and Krackhardt, 2008), rather they are strongly influenced and shaped by social connections (Obembe, 2006). In the research on friendship network formation, the network structures of primary concern include homogeneity, reciprocity, transitive triples, cyclic triples, and geometrically-weighted edgewise shared partnerships (GWESP) parameters (Duarte-Barahona et al., 2020).

Reciprocity, which may be based on gender, personality similarity, academic achievement, psychological age, and intelligence (Bonney, 1946), is a network formation process that does not require complex coordination activities (Heidler et al., 2014). Reciprocity can be expressed as a person who is more likely to be attracted by others who are similar to them (Byrne, 1971). In a study on the role of personality traits in the formation of friendship networks, this reciprocity was referred to as the personality homogeneity effect (McPherson et al., 2001). Reciprocity is not only similarity attraction, but also involves a binary mixed selectivity (Goodreau et al., 2009), including similarity of choice (assortative mixing) and the opposite trend (disassortative mixing), such as the complementary response in human relationship theory (Horowitz et al., 2006). Compared with reciprocity, the emergence of triadic closure reflects a more complex process of network formation; triads tend to close, leading to a nuclei of cliques (Heidler et al., 2014). For example, in directed networks, the introduction of GWESP parameters can effectively identify the trend of triadic closure based on transitivity or cyclicity (Hunter, 2007). Irrespective of the type of triadic closure, it is the basis for the emergence and formation of small groups. Whereas transitive closure corresponds with hierarchical relations, cyclicity is more affiliated with equality and collaboration.

The formation of a friendship network may be based on individual attributes, but it may also be based on the structure of the network itself (i.e., endogenous tie formation processes). Reciprocity can be regarded as a basic network structure formed by the friendship network relationship (Kitts and Leal, 2021). Similarly, friendships are transitive, which means individuals in the class are in some triangular structures. From this theoretical background, we issue the following two hypotheses that concern the influence of endogenous tie formation process on the formation of friendship network:

**Hpy.6.** Reciprocity (based on personality traits) would have a significant impact on the formation of a friendship network.

**Hpy.7.** Triad closure would have a significant impact on the formation of a friendship network.

### The present study

In the present study, we investigated the network clustering and group characteristics of college students’ friendships through individual social attributes (i.e., individual characteristics) and network structural factors (i.e., endogenous tie formation processes). We focused on personality traits, interpersonal self-efficacy, reciprocity, and triples. Four steps were conducted to demonstrate the network clustering, group characteristics, and formation process of college students’ friendship networks in the study.

Firstly, we developed of a scale to measure Chinese college students’ interpersonal self-efficacy. Secondly, we investigated the influence of personality traits on the formation of friendship networks and their influence on an individual’s position in the network. Thirdly, we analyzed the influence of interpersonal self-efficacy on the scale of college studentsed of a scale to measure Chinese college students’ interpersonal self-efficacy. Secondly, we investigated the influence of personality by using the survey results of interpersonal self-efficacy scale. Essentially, the above steps explored the network clustering and group characteristics of college students’ friendship relationships from two aspects: personality traits and interpersonal self-efficacy. Finally, we investigated the formation process of college students’ friendship networks from the perspective of network structure and verified all the steps by constructing an exponential random graph model (ERGM).

## Data variables and methods

### Data collection

The data have been collected in September and October 2021 at Inner Mongolia University, Hohhot. Data collection is divided into two stages. Two surveys were conducted in first stage. 260 students participated in the survey (71.60% women; mean age [*M*_age_] = 22.47, standard deviation [SD] = 2.34; 32 freshmen, 56 sophomores, 43 juniors, 76 seniors, and 53 graduate students) in the first administration. The main purpose of this survey is to complete the initial questionnaire of interpersonal self-efficacy, which had a total of 40 items (coded Q1--Q40). In the second administration, 146 college students participated in the survey (74.00% women; *M*_age_ = 20.50, *SD* = 1.96; 29 freshmen, 36 sophomores, 32 juniors, 23 seniors, and 26 graduate students). Participants were asked to complete the Interpersonal self-efficacy scale, which is based on the initial questionnaire, including 20 items^2^.

In the second stage, 86 students in a sophomore class at the School of Economics and Management at Inner Mongolia University (*M*_age_ = 20.35; *SD* = 3.21). Participated in the survey. Four students withdrew from the study due to special circumstances, resulting in a sample of 82 participants (25 boys and 57 girls). Participants were asked to provide information on demographic characteristics, class friendship network, and personality traits [we used the International Professional Edition of the Dominance, Influence, Steadiness, and Conscientiousness personality test (DISC Scale)^3^]. Demographic variables included gender, age, parentlity test (, Influence, Stsocioeconomic status. To measure class friendship network, we used the nomination method; a friendship relationship matrix from the names of the 82 students in the class was created. The question was, “which of the following students are your friends or would you like to be friends with?”

Through two stages data collection, we obtained a class friendship network data including 82 students, personality traits and interpersonal efficacy data.

### Date description

UCINET 6.0 software was used to visually describe the friendship network structure of the participants. There is strong group isolation in the network (clear and identified cliques). The structure of the network can be checked in more detail by calculating the basic structural parameters described in Table 1.

**TABLE 1:** Basic structural parameters of friendship network.

Network structure parameters	Value
Actors	82
Average degree	5.49
Connectedness	1.00
Proportion of mutualities	0.46
Average path length	2.61
Average clustering coefficient	0.46
Indegree-centralization	13.14%
Outdegree-centralization	15.64%
Modularity	0.67

**FIGURE 1:**
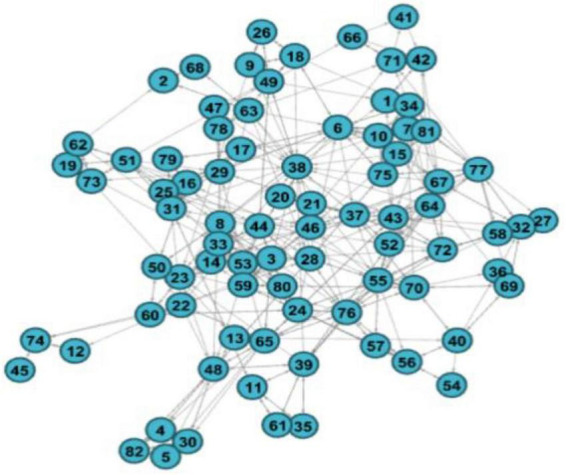
Random graph of friendship network.

The network contained 82 students with an average of 5.49 network ties. The connectedness value of 1.00 shows that every student in the class had a connection, and there is no isolated point. The average path length from one student to another was 2.61; nevertheless, the clustering coefficient is high, with a mean value of 0.46. The clustering coefficient shows that the class displays a clear tendency toward triadic closure, although a test for the exact structural arrangement requires a probabilistic rather than a descriptive approach, which will be provided in the ERGM. The in-degree centralization in the friendship network is higher than the outdegree centralization, which means that the inequality of popularity (receiving ties) is higher than the inequality of friendship activity (sending ties). The modularity indicating a division into dense subsets has a value that is generally regarded as high, which was 0.67. This corresponds to the impression of the visualization, which exhibits clearly identifiable cliques (Figure 1).

The DISK scale has 40 items and each item has four response options. Based on their scores, the participants were classified as follows: three (3.66%) Dominance type; 27 (32.93%) Influence type; 34 (41.46%) Steadiness type; 15 (18.29%) Compliance type; and three (3.66%) Chameleon type.^4^ 82 participants completed the Interpersonal self-efficacy scale (*M_score_* = 54.4, *SD_score_* = 9.9), which includes the following five dimensions: communication efficacy (*M* = 16.18, *SD* = 2.97), observation and listening efficacy (*M* = 11.02, *SD* = 2.36), altruistic efficacy (*M* = 7.93, *SD* = 1.79), affinity efficacy (*M* = 10.93, *SD* = 2.89), and emotion control efficacy (*M* = 8.24 *SD* = 1.90).

### Variable selection

In the friendship network, A chooses B as a friend, and B often chooses A as a friend. Friendship relationships are often characterized by reciprocity, which can be regarded as a basic network structure formed by the friendship network relationship. Similarly, friendships are transitive. If A chooses B as a friend and B chooses C as a friend, the probability of A and C becoming friends will increase. Among triad closures, there are different structures, such as transitive triples and cyclic triples. Previous studies mostly chose GWESP (transitive path closure of multiple 2-paths) based on triad closure. Considering the scale of the friendship network and the more interactive behavior of students, we chose balance in the cyclic triplet as the structural factor to predict the formation of a friendship network. In the core-periphery analysis, some members are in the center of the friendship network. Therefore, the popularity (in-degree effect) and activity (out-degree effect) were also included in the structure. At the same time, the edges of the network structure are considered, and the structural elements in the ERGM are shown in Table 2.

**TABLE 2:** The structural elements in the exponential random graph model (ERGM).

Network structure	Name in Statnet	Figure	Explanation
Arc	Edges	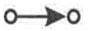	Benchmark tendency of relationship formation
Reciprocity	Mutual	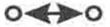	This variable is often positive, indicating that reciprocity is likely to be observed for positive impact networks.
Popularity (in-degree)	Idegree	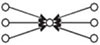	The negative popularity parameter shows that most actors have a similar level of popularity (the network is not in-degree centered).
Activity (out-degree)	Odegree	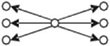	The negative activity parameter shows that most actors have a similar level of activity (the network is not out-degree centered).
Cyclic closure (Triangle structure)	Balance	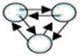	The positive effect here shows that there is a high degree of closure or multiple triangular clusters in the network.
Transitivity (Transitive path closure)	GWSEP	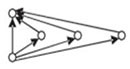	Number of structures in which two individuals have common partners.

The calculation formula of gwesp, edges, idegree, odegree, and mutual can refer to Wu et al. (2020).

Individual characteristics mainly include personality traits and interpersonal self-efficacy (abbreviated as Ises). The impact of these two variables on the formation of friendship networks has been widely discussed in the literature. Gender factors are also considered as they cannot be ignored in this context.

### Method selection

To test the hypothesis 1 and hypothesis 4 that different personality traits and interpersonal self-efficacy would have a significant impact on the scale of the friendship network, we used a one-way ANOVA and *t*-test. The results are provided in Table 3. To test the hypothesis 2 and hypothesis 5 that different personality traits and interpersonal self-efficacy would have a significant impact on the position of the friendship network, we used core-periphery analysis by using UCINET 6.0. In order to test hypothesis 3, we used analysis of cohesive subgroups. To test the hypothesis 6 and hypothesis 7, we used Exponential Random Graph Model (ERGM).

**TABLE 3:** Relationship density within and between different cohesive subgroups.

Subgroups	1	2	3	4	5	6	7	8
1	0.289	0.100	0.027	0.040	0.030	0.055	0.010	0.030
2	0.010	**0.267**	0.036	0.000	0.040	0.000	0.010	0.000
3	0.027	0.009	**0.355**	0.027	0.018	0.017	0.027	0.018
4	0.030	0.070	0.018	**0.500**	0.020	0.091	0.000	0.070
5	0.040	0.030	0.055	0.050	**0.467**	0.045	0.060	0.100
6	0.018	0.018	0.033	0.073	0.027	**0.373**	0.009	0.000
7	0.010	0.010	0.036	0.010	0.040	0.036	**0.222**	0.030
8	0.040	0.000	0.018	0.080	0.070	0.036	0.010	**0.356**

**FIGURE 2:**
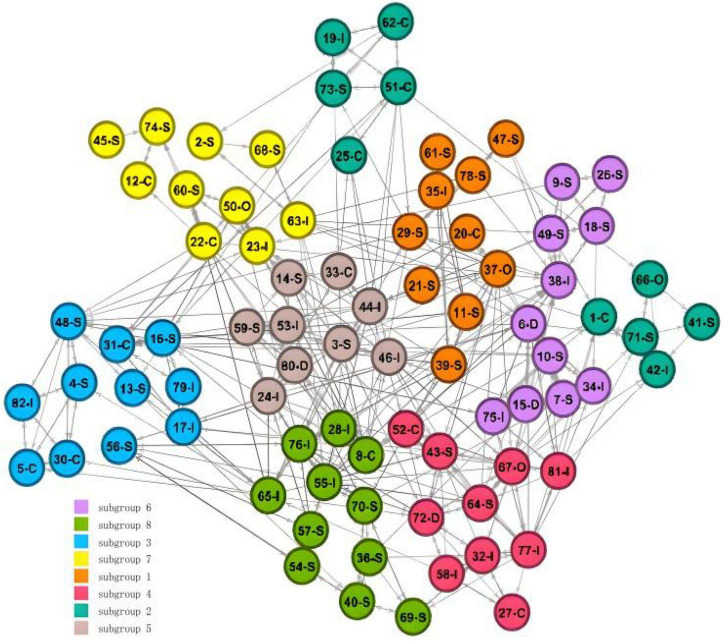
Analysis of cohesive subgroups formed by friendship network. Numbers represent the number of nodes (each individual). D, dominance traits; I, influence traits; S, steadiness traits; C, compliance traits; O, chameleon traits.

The results in table are calculated according to the cohesive subgroup in Figure 2, and the personality traits of individuals within each cohesive subgroup are shown in in Figure 2. The bold values refer to relationship density within cooperative subgroup. Non-bold values refer to relationship density between cooperative subgroup.

### Model setting

The ERGM was applied to analyze the class friendship network and test our hypothesis. An ERGM (also called p-star) is based on a probabilistic approach that treats networks as realizations of random variables, with an exponential family distribution (Lusher et al., 2013). ERGMs can be used to model the global network structure based on the local rules of tie selection. Local parameters, such as homophily and transitivity can be combined with a multivariate model fitted to the empirical network structure using a Markov Chain Monte Carlo (MCMC) procedure. To achieve this result, we used the well-established ERGM module in the “Statnet” package for R to build an ERGM of the friendship network and test the hypothesis.

The distribution of the random term Y in the ERGM can be expressed as


(1) $$P\left(Y=y|\theta \right)=\frac{1}{k\,\left(\theta \right)}\exp\left\{\sum_{H}\theta_{H}g_{H}\left(y\right)\right\}$$

We define *Y*_*ij*_ as a random variable for the tie between individuals i and *j*(*Y*_*ij*_ = 1 When i and j share a relationship, and *Y*_*ij*_ = 0 otherwise). These ties can be represented as a N × N matrix (*N* is the number of individuals) called Y, which represents a random network set of class friendship networks. We specify *y*_*ij*_ as the observed value of *Y*_*ij*_, and y is the matrix of all observed ties. Here *k*(θ) is a normalizing quantity to ensure that (1) is a proper probability distribution (Robins et al., 2007; Hunter et al., 2013). Term θ_*H*_ is the parameter and *g*_*H*_(*y*) is the network statistic corresponding to configuration. θ_*H*_ is the main parameter concerned in the follow-up empirical research. The influence of different factors on the formation of friendship networks is judged by the significance and value of θ_*H*_. *H* refers to all factors that may affect the formation of network y (Robins et al., 2007), generally including the endogenous structural factor a (edge, triangular structure, star structure, etc.), the nodes attribute factor b (personality and self-efficacy of each individual in this study), and the other network factor c, and *abc* ∈ *H*. Therefore, the ERGM can be written as follows:


(2) $$P\left(Y=y|\theta \right)=\frac{1}{k\,\left(\theta \right)}\exp\left\{\theta_{a}g\left(y\right)+\theta_{b}g\left(y,X\right)+\theta_{c}g\left(y,Z\right)\right\}$$

where *g*(*y*), *g*(*y*,*X*), and *g*(*y*,*Z*) are model variables that indicate any set of network statistics calculated by *y* and hypothesized to affect the probability of network formation. The three terms represent the three effects: *g*(*y*) is the pure structural effect, which is the network statistic corresponding to endogenous network structural configurations; *g*(*y*,*X*) represents the socially selective effect, which is the network statistic corresponding to the node attributes X; *g*(*y*,*Z*) is the relational embeddedness effect, which is the network statistic corresponding to the other network Z (not considered here). θ_*a*_, θ_*b*_, and θ_*c*_ are unknown parameters that need to be estimated.

Specifically:

Model 1 (Basic model): Only edges were considered.


(3) $$P\left(Y=y|\theta \right)=\frac{1}{k\,\left(\theta \right)}\exp\left\{\theta_{a\,1}\cdot Edges\right\}$$

Model 2 (Structure model): Endogenous structural factors were considered, including edges, idegree, odegree, mutual balance and Gwesp.


(4) $$P\left(Y=y|\theta \right)=\frac{1}{k\,\left(\theta \right)}\,\exp\,\left\{\begin{array}{c} \theta_{a\,1}\cdot E\,d\,g\,e\,s+\theta_{a\,2}\cdot I\,d\,e\,g\,r\,e\,e+\theta_{a\,3}\cdot O\,d\,e\,g\,r\,e\,e+\\ \theta_{a\,4}\cdot M\,u\,t\,u\,a\,l+\theta_{a\,5}\cdot B\,a\,l\,a\,n\,c\,e+\theta_{a\,6}\cdot G\,W\,E\,S\,P \end{array}\right\}$$

Model 3 (Attribute model): Individual attributes were considered, including personality traits and interpersonal self-efficacy (abbreviated as Ises).


(5) $$P\left(Y=y|\theta \right)=\frac{1}{k\,\left(\theta \right)}\,\exp\left\{\theta_{b\,1}\cdot P\,e\,r\,s\,o\,n\,a\,l\,i\,t\,y+\theta_{b\,2}\cdot I\,s\,e\,s+\theta_{3\,b}\cdot G\,e\,n\,d\,e\,r\right\}$$

Model 4 (Actor attribute model): Both endogenous structural factors and individual attributes were considered.


(6) $$P\left(Y=y|\theta \right)=\frac{1}{k\,\left(\theta \right)}\,\exp\,\left\{\begin{array}{c} \theta_{a\,1}\cdot E\,d\,g\,e\,s+\theta_{a\,2}\cdot I\,d\,e\,g\,r\,e\,e+\theta_{a\,3}\cdot O\,d\,e\,g\,r\,e\,e\\ +\theta_{a\,4}\cdot M\,u\,t\,u\,a\,l+\theta_{a\,5}\cdot B\,a\,l\,a\,n\,c\,e+\theta_{a\,6}\cdot G\,W\,E\,S\,P\\ +\theta_{b\,1}\cdot P\,e\,r\,s\,o\,n\,a\,l\,i\,t\,y+\theta_{b\,2}\cdot I\,s\,e\,s+\theta_{b\,3}\cdot G\,e\,n\,d\,e\,r \end{array}\right\}$$

## Results

### Personality traits and individuals’ friendship network scale

We used a one-way ANOVA and *t*-test of sample differences in means to test hypothesis 1. The results are provided in Table 4.

**TABLE 4:** Average network scale and test results of college students with different personality traits.

Reference group	Personality type	Average network scale (in-degree)	Difference in means	*t*	*p*
Dominance type (8.67)	Influence	6.44	2.23	1.136	0.266
	Steadiness	4.47	4.20**	2.458	0.019
	Compliance	5.00	3.67*	1.853	0.082
	Chameleon	7.67	1.00	0.243	0.820

*p < 0.1, **p < 0.05.

The one-way ANOVA of different personality types on individual in-degree centrality was significant (*F* = 3.10, *df* = 81, *p* = 0.02), which means that personality had a significant impact on the scale of the friendship network. Specifically, personality differences affected how many people chose them as friends. However, the influence of different personality traits on individual out-degree centrality was not significant (*F* = 1.90, *df* = 81, *p* = 0.12), which means that personality differences had no significant effect on how many other people individuals chose to be friends with. Furthermore, we conducted a *t*-test on the mean difference of friendship network scale (only testing the in-degree centrality) of different personality types and found that there was no significant difference between the dominance personality and the influence personality, while the scale of friendship network between the dominance personality and the steadiness personality and compliance personality were very different. The individual friendship network of the dominance and the influence personality types is larger. Hypothesis 1 had been verified.

### Personality traits and individuals’ friendship network clustering

We can use the analysis method of cohesive subgroups to test the hypothesis 2. As previously assumed, we hypothesized that the friendship network would show different small groups, and individuals with similar personality traits (homogeneity) would be more likely to become friends. Through the clearly identifiable cliques in Figure 1, the cohesive subgroup analysis is carried out based on factions, and finally it is divided into eight factions. The final proportion of correct answers was 0.90. The results of the factions are shown in Figure 2, while Table 3 shows the relationship density within and between different cohesive subgroups.

The results of these analysis do not seem to verify the hypothesis 2 from the perspective of the personality traits of different members within the group (this will be further confirmed in the ERGM). On the contrary, individuals with complementary and different personality traits were more likely to become friends, which indirectly verifies the existence of the complementarity effect. More importantly, from the density of friendship networks within each subgroup, the cohesive subgroup with more dominance or influence personality traits had a higher internal density. For example, the network densities within subgroups 4 and 5 were 0.500 and 0.467, respectively. The internal density of the cohesive subgroup with more steadiness and compliance personality traits was relatively low. For example, the internal network densities of subgroups 7 and 2 were 0.222 and 0.267, respectively.

### Personality traits and individuals’ position in friendship network

**FIGURE 3:**
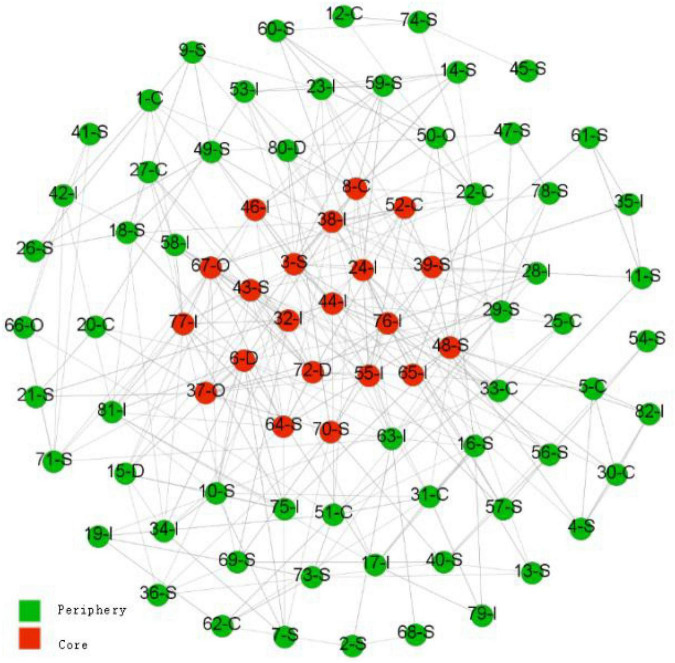
Core-periphery analysis of friendship network (personality traits). Numbers represent the number of nodes (each individual). D, dominance traits; I, influence traits; S, steadiness traits; C, compliance traits; O, chameleon traits.

Although the friendship network of the class presents the characteristics of small groups, the network density within each subgroup is different, and the cohesive subgroups that predominantly comprise individuals with dominance or influence personality traits are large. Therefore, we believe that in the class friendship network, there is likely to be a core-periphery distribution. The core-periphery analysis (missing value) was used to test the hypothesis 2 that personality traits would affect the individuals’ position in the friendship network and the results are shown in Figure 3.

The final fitting value of the core-periphery analysis (missing value) was 0.260 (directed network), which is not very high. This may be related to the small groups in the friendship network. However, from the analysis results, it can be found that dominance and chameleon personality types are in the center of the class friendship network, followed by individuals with the influence personality type. Although individuals with the steadiness personality accounted for the largest proportion in the class, their proportion in the center of the friendship network was very low. The same situation occurs with the compliance personality; individuals with steadiness and compliance personalities were more on the periphery of the friendship network. The influence type can be characterized as extraverts, which provides support for our hypothesis 3 that extraverts would be in the center of the friendship network.

### Interpersonal self-efficacy and individuals’ friendship network scale

The hypothesis 4 was tested using correlation coefficient (between interpersonal self-efficacy score and point degree centrality). The results are presented in Table 5.

**TABLE 5:** Correlations between interpersonal self-efficacy and friendship network scale.

	In-degree	Out-degree	Between
Ises	0.51***	0.44***	0.47***

***p < 0.001. Pearson correlation coefficient.

Through the correlation coefficient, it was found that interpersonal self-efficacy had the strongest relationship with in-degree centrality, indicating that individuals with high interpersonal self-efficacy were more popular with others. The correlation coefficient between interpersonal self-efficacy and betweenness centrality indicates that individuals with high interpersonal self-efficacy not only had a relatively large-scale friendship network, but were also more likely to become intermediaries in the process of making friends. Hypothesis 4 had been verified.

### Interpersonal self-efficacy and individuals’ position in friendship networks

**FIGURE 4:**
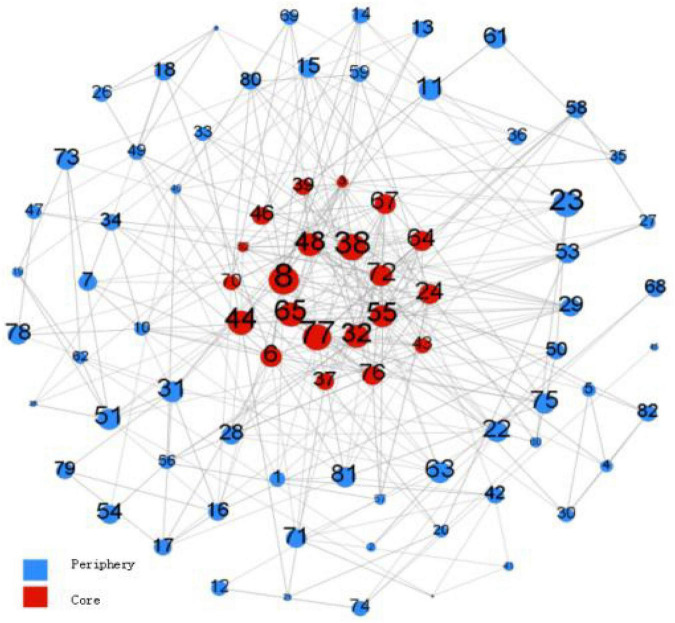
Core-periphery analysis of friendship network (interpersonal self-efficacy). The number represents the number of nodes (each individual); the larger the circle, the higher the interpersonal self-efficacy.

We hypothesized that people with higher interpersonal self-efficacy would be more likely to be in the center of the friendship network, while people with lower self-efficacy would be at the edge of the network. The core-periphery analysis was used to test hypothesis 5 and the results were shown in Figure 4.

The results show that individuals with higher interpersonal self-efficacy were more likely to be in the center of the friendship network, in which the relationships of the core members is close, while the relationships of the members at the periphery of the friendship network are more distant or detached. Although interpersonal self-efficacy will have a certain impact on whether members are in the center of the friendship network, this is not absolute. As hypothesis 3 showed, personality traits also affect members’ positions in friendship networks. In addition, the formation of a friendship network is also affected by structural factors, which requires us to analyze it in combination with more research.

### Interactive effect of personality traits and interpersonal self-efficacy on the position in friendship networks

We found that members with dominance and influence personality traits or members high interpersonal self-efficacy were more likely to be in the center of the friendship network in hypothesis 3 and hypothesis 5. Is there a relationship between personality traits and interpersonal self-efficacy? To address this question, we conducted a *t*-test to compare the average scores for interpersonal self-efficacy with the different personality traits. The results are presented in Table 6.

**TABLE 6:** Mean difference results comparing interpersonal self-efficacy scores of different personality traits.

Reference group	Personality trait	Interpersonal self-efficacy (mean)	Mean deviation	*t*-value	*p*-value
Dominance type (61.33)	Influence	58.48	2.85	0.38	0.70
	Steadiness	50.88	10.45	2.03	0.05
	Compliance	52.80	8.53	2.60	0.02
	Chameleon	48.67	12.67	1.19	0.30

From the results in Table 6, the average score of interpersonal self-efficacy for dominance personality traits was the highest, which was significantly different from the average score for interpersonal self-efficacy of steadiness and compliance personality traits. In other words, members with steadiness and compliance personality traits had relatively lower interpersonal self-efficacy. To a certain extent, this demonstrates that there exists a joint effect or positive interactive effect on the influence of personality traits and interpersonal self-efficacy on the scale and position of an individual’s friendship network. Individuals with dominance and influence personality traits also had relatively high interpersonal self-efficacy.

### The influence of network structure on the formation of friendship network

**FIGURE 5:**
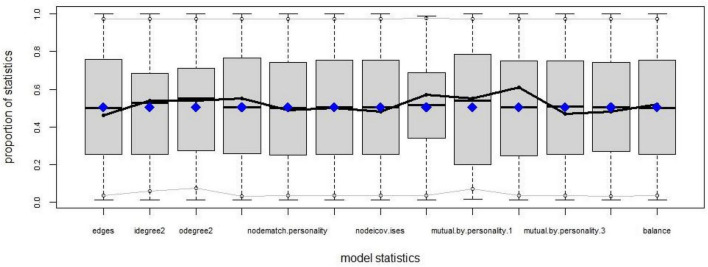
Fitting effect of the exponential random graph model.

To test hypothesis 6 and Hypothesis 7, we used ERGM to analyze how endogenous structural factors (including edges, mutual, idegree, odegree, balance, and GWESP) affect the formation of friendship networks. At the same time, individual attributes (including personality traits and interpersonal self–efficacy) were considered in the REGM. Model 2 only considered the influence of endogenous structural factors. Model 3 only considered the influence of individual attributes. Model 4 comprehensively considered the common influence of endogenous structural factors and individual attributes. Using the “Statnet” package of R software, the estimation results are shown in Table 7, and the fitting results are shown in Figure 5.

**TABLE 7:** ERGM estimated results for friendship network formation.

	Model 1	Model 2	Model 3	Model 4
**Network structure effect**				
Edges	−2.62***(0.05)	−4.06***(0.18)		−3.32***(0.48)
Idegree		0.86**(0.41)		2.10***(0.48)
Odegree		0.36*(0.47)		1.61***(0.52)
Mutual(personality.D)		−3.27***(0.94)		−7.57***(1.25)
Mutual(personality.I)		−2.75***(0.75)		−6.65***(1.18)
Mutual(personality.S)		−2.58***(0.74)		−6.27***(1.18)
Mutual(personality.C)		−2.63***(0.75)		−6.46***(1.19)
Balance		1.31***(0.11)		0.24***(0.03)
GWESP(transitivity)		0.12***(0.02)		
**Individual sociality effect**				
Homophily(personality.D)			1.44(1.13)	
Homophily(personality.I)			0.15(0.16)	
Homophily(personality.S)			−0.13(0.13)	
Homophily(personality.C)			−0.24(0.28)	
Ises			−0.02***(0.00)	
Gender			−0.13*(0.09)	−0.03(0.09)
Personality				−0.01(0.09)
Receiver(ises)				0.02**(0.09)
Sender(ises)				0.01(0.01)
*AIC*	−3626.77	−4550.34	−3541	−4280.71
*BIC*	−3619.97	−4482.33	−3521	−4192.30
Log likelihood	1814.39	2285.71	1773.73	2153.36

D, dominance traits; I, influence traits; S, steadiness traits; C, compliance traits; O, chameleon traits; Ises, interpersonal self-efficacy. *p < 0.05, **p < 0.005, ***p < 0.001.

From the Figure 5, the overall fitting effect of the model is good except that the out-degree is poor. The network structure factors shown in Model 2, such as in-degree, out-degree, reciprocity, and closed triples, have a significant impact on the formation of friendship networks. The network individuals’ attributes shown in Model 3, such as interpersonal self-efficacy and gender, also have a significant impact.

#### Homogeneity and individuals’ friendship networks

Hypothesis 2 showed that members with different personalities were more likely to form small groups than members with similar personalities, which suggests that the reciprocity of a friendship network is disassortative mixing, also known as the complementary response of interpersonal relationships. From the results of the homogeneity effect in Model 3, the regression coefficients of four types of personality traits were 1.44, 0.15, −1.03 and −0.24, respectively, which were not significant and did not show an intragroup homogeneity effect. The reciprocity effect results of Model 4 showed that the reciprocity coefficients based on the same personality attributes were all negative and significant (*P* < 0.001). Reciprocity and homogeneity analysis further verified hypothesis 2. While, the hypothesis 6 based on the reciprocity of personality similarity had not been tested. Model 4 showed that interpersonal self-efficacy had a significant positive effect on friendship networks, which further demonstrated the conclusion of Hypothesis 4.

#### Triad closure and individuals’ friendship networks

In model 2, we add gwesp and balance. The results showed that the odds for a tie to close a triangle was over 1.13 (e0.12 = 1.13) times the odds for the tie not to close a triangle (*P* < 0.001), which provided support for transitivity in friendship networks. We only chose a balance in the triangular structure in Model 4. The regression coefficient of balance was 0.24 and was significant, which shows that the tie to close triangle was a path closure trend of two-way cycle in friendship networks, and triangles often appear easily in dense areas of the network. Therefore, Hypothesis 7 was verified. The finding also demonstrates the phenomenon of group clustering in friendship networks. It shows that the network is often closed in the structure of mutual circulation. The formation of friendship groupings appears to be done based on closeness.

#### In- and out-degree and individuals’ friendship networks

Regarding popularity and activity, these effects represent tendencies for centralization in the in-degree and out-degree distributions, respectively. The in-degree parameter estimate was 2.19 and significant, indicating that under the control of other factors, there were class members who were particularly popular with other students in the class. Similarly, the out-degree parameter estimate was 1.73 and significant, indicating that there was an expansion trend in the friendship network. In-degree and out-degree effects show that there is a core-periphery phenomenon in the class friendship network, which can be further explained by the sender and receiver effect. The sender/receiver effect measures the degree to which actors with a specific attribute send/receive more ties compared to others in the network. The coefficient of receiver effect (interpersonal self-efficacy) was 0.03 and significant, which shows that individuals with high interpersonal self-efficacy are more welcome, can harvest more friendships, and are easy to be in the center of the friendship network. This conclusion also further verified the hypothesis 5. The coefficient of the sender effect (interpersonal self-efficacy) was not significant.

The results of ERGM (from model 2 to model 4) were used to verify hypothesis 6 and Hypothesis 7. That is, the results verified the impact of reciprocity and triangular structure on the formation of friendship networks. At the same time, based on the homogeneity of personality traits, in-degree, out-degree, receiver effect and sender effect, it also provides further evidence for hypothesis 2, hypothesis 4 and hypothesis 5.

## Discussion

### General discussion

College friendships are an early source of social capital in adulthood. Adolescents with more friends tend to enjoy stronger social support and have higher social status in their groups, which is beneficial to their future careers (Shi and Moody, 2017). The formation of a friendship network is a dynamic selection process, and previous studies have offered two major perspectives to understand friendship formation: (1) the role of individual characteristics, such as personality traits and gender (Gest et al., 2002; Lewis, 2013), and (2) incorporating endogenous tie formation processes such as reciprocity and transitivity (Goodreau et al., 2009; Wimmer and Lewis, 2010). Based on social cognitive theory and social network theory, this study emphasized the formation of college students’ friendship networks, network clustering, and group characteristics. In the four studies, we analyzed and commented on different aspects, including the measurement of interpersonal self-efficacy; personality traits and friendship networks; interpersonal self-efficacy and friendship networks; and network structure (endogenous process) and friendship network. We integrated the two different theoretical perspectives and combined the network method with individual characteristics to analyze the formation process of a friendship network. The results show that both individual characteristics and network structure (endogenous processes) have an impact on friendship. These processes interact and produce complex effects.

Development of the interpersonal self-efficacy scale was a basic preparation study. To more accurately reflect the impact of interpersonal self-efficacy on the establishment of friendship relationships, a scale of interpersonal self-efficacy was developed for the purpose of the present study and was based on previous research. This scale comprised five dimensions of interpersonal self-efficacy. The results found that college students’ friendship networks existed in the form of small groups, which were largely based on differences in personality traits among friends (Selden and Goodie, 2018). The influence of personality traits was not only related to the scale of individual friendship networks, but also the position of individuals with different personality traits in the network (Goodreau et al., 2009; Hullman et al., 2010). The position in the network directly determines their importance in class friendship relationships. The same conclusion occurred in interpersonal self-efficacy, which demonstrated the impact of another aspect of individual characteristics on friendship network. Similarly, differences in interpersonal self-efficacy affected the scale of the network and the position of the individual in the friendship networks (Locke and Sadler, 2007). Thus, the influence of personality traits and interpersonal self-efficacy on friendship networks showed that individuals who have traits associated with extraversion (e.g., the influence type) and high interpersonal self-efficacy tended to have large scale networks and were in the center of the friendship network (Zell et al., 2014; Siciliano, 2016).

Finally, ERGM systematically explained the formation of a friendship network based on the consideration of network structure (i.e., undeveloped tie formation processes) and individual characteristics. Our analysis revealed evidence that both selective mixing and triad closure operated across a wide range of sociodemographic settings to structure the process of mutual friendship formation (Horowitz et al., 2006; Goodreau et al., 2009). Personality traits and interpersonal self-efficacy generated strong assortative mixing and within-category triad closure. Specifically, the heterogeneity effect of personality traits explained the formation mechanism of small groups, and the homogeneity effect of self-efficacy also explained the core periphery distribution in friendships to a certain extent.

### Implications

This study provides a unique paradigm. Based on social cognitive theory, personality traits would affect behavior choice; for example, dominance traits can predict dominance behavior. Here we focused on interpersonal behavior as it relates to friendship networks. Persons who possess complementary personality traits tend to be more satisfying (Kiesler, 1983), which means interactants who are dissimilar in their personality traits would feel more positively about their interactions and about each other. In the small group formed in the friendship network and ERGM, interpersonal complementarity reflects the above view. This shows that in friendship, personality complementarity has a stronger impact on attraction and cohesion than similarity (O’Connor and Dyce, 1997). The findings regarding interpersonal self-efficacy supported the transmission effect: individuals with higher interpersonal self-efficacy have a closer relationship with their peers, which predicts greater dyadic satisfaction. The implication of this result is that people with higher interpersonal self-efficacy are more likely to establish closer friendships than those with lower interpersonal self-efficacy (Locke and Sadler, 2007). A new phenomenon has emerged in the study of the transmission effect of interpersonal self-efficacy, which we temporarily call collective efficacy. It was found that a dynamic friendship process is likely to form common collective efficacy. Individuals in the center of a friendship network tend to have high interpersonal self-efficacy, and they tend to have similar collective efficacy (Myers et al., 2004). As previously mentioned, this type of transactional cycle is a key construct of both interpersonal and social cognitive theories. Elucidating verbal behaviors (e.g., communication and expression behavior) and non-verbal behaviors (e.g., altruistic behavior and affinity behavior) through which partners communicate collective efficacy will be an interesting challenge for future research.

The two core individual difference variables in social cognitive theory are expectation and self-efficacy (Mischel, 1973). Based on the core-periphery analysis, it was found that there was a relationship between two individual characteristics affecting friendship: personality traits and interpersonal self-efficacy. The results of Studies 2 and 3 show that individuals with dominance and influence personality traits are more likely to be at the center of the network, which also occurs in individuals with high interpersonal self-efficacy. Therefore, we believe that personality traits and self-efficacy have interactive effects on the formation of friendship networks to some extent. Personality traits are relatively stable, and interpersonal self-efficacy changes in different environments. Some studies have shown that interpersonal self-efficacy plays an intermediary role in students’ personality traits and social skills (Shamsi and Amirianzadeh, 2017).

Social network theory emphasizes the endogenous formation process of a specific network (An, 2022), such as reciprocity (i.e., the tendency to reciprocate friendship ties), transitivity (i.e., the tendency to be a friend of a friend), and preferential attachment (i.e., the tendency to be a friend of a popular person). However, individual characteristics and behavior may in turn affect the choice of network partners and promote the establishment of network relationships, and personality traits and self-efficacy that highlight individual differences in social cognitive theory have also been widely supported in the establishment of friendships (Lewis, 2013). This study combines two theoretical perspectives. In the ERGM, which comprehensively considers the combination process of individual characteristics and network endogeneity, it is found that the hypothesis of friendship relationships based on personality reciprocity (i.e., homogeneity) was not tenable. The establishment of a friendship relationship verifies the complementary response of personality traits. In the analysis of the receiver effect (interpersonal self-efficacy), sender effect (interpersonal self-efficacy), popularity (in-degree effect), and activity (out-degree effect), it was demonstrated that there are very popular individuals in the class friendship network, indicating that there is a core-periphery distribution. When examining the influence of the triangular structure, it was found that the transitivity of the friendship relationship is more a two-way circular relationship, that is, it is closer than GWESP (transitivity).

### Limitations and future directions

Although we studied the formation, network clustering, and group characteristics of college studentsmentary response of pefrom two theoretical perspectives, there are some limitations that deserve mentioning. Starting from social cognitive theory, we emphasized the influence of individual characteristics, including personality traits and interpersonal self-efficacy, on the formation of friendship, but the factors considered are far from sufficient. For college students, differences in family background and academic achievement can shape the formation of friendships, which hides the influence of reputation (An and Mcconnell, 2015; An, 2022). This kind of status-grade difference from social exchange theory also implies the formation mechanism of friendship relations. For example, friendship relations are more likely to develop from persons with lower status to those with higher status, rather than the opposite (Gravatt et al., 2013). These potential areas of influence were not investigated in the current study.

Another limitation of the current work is that although we found suggestive evidence that personality traits and interpersonal self-efficacy may have interactive effects on the formation of friendship networks, causal relationships cannot be determined. Moreover, there is no clear explanation of whether interpersonal self-efficacy mediates the relationship between personality traits and interpersonal communication. We find that it is easier to explain the agglomeration of friendships on a small scale in the triangular structure. We try to introduce GWESP (transition) and geometrically weighted dyad-wise shared partners (GWDSP; two paths), but the overall fitting effect of the model is not good in Model 4.

Finally, we believe that the establishment of a friendship relationship is a dynamic process, and the friendship relationship is constantly changing. Cross-sectional data only captures one point in time, which limits our ability to capture any change in friendship networks over time. Future research should include a longitudinal survey design to investigate whether the variables included in the present study are stable factors for the long-term maintenance of friendship, as well as factors such as academic achievement and family socioeconomic status. Moreover, among many individual characteristics that affect friendship, some often do not work alone, and there may be interaction effects between different variables, as we mentioned regarding personality traits and interpersonal self-efficacy. To do so will provide a more comprehensive understanding of the formation of friendships. Similarly, any network has its endogenous tie-formation processes, which reflect the influence of network structure factors. More triangular structures introduced such as GWESP (transition) and GWDSP (two paths) will more clearly reveal the trend of friendship establishment based on transitive or circularity in future studies.

## Data availability statement

The datasets presented in this study can be found in online repositories. The names of the repository/repositories and accession number(s) can be found in the article/Supplementary material.

## Author contributions

DY conceived of the presented idea. DY and XY designed the methods of data collection and performed the data analysis. DY and HZ wrote the first draft of the manuscript and commented on and revised the drafts. All authors contributed to the article and approved the submitted version.

## Footnotes

1 The types and explanations of five factor model personality traits are given in Appendix 1.

2 The preparation and measurement of the questionnaire and the division of different dimensions will be given in the Appendix 1.

3 Disc scale is an international general personality test scale, which is similar to the big five personality test. Disc personality test consists of 24 groups of adjectives describing personality traits. Each group contains four adjectives. These adjectives are selected according to Dominance (D), Influence (I), Steadiness (S), Compliance (C), four measurement dimensions and some interference dimensions. The subjects were asked to choose one of the adjectives that was most suitable for themselves and least suitable for themselves. Finally, individuals will be divided into different personality types according to their scores.

4 Dominance (The director): adventurous, competitive, bold, direct, decisive, innovative, persistent, problem solver, and self-motivated; Influence (The interaction): charming, confident, persuasive, enthusiastic, inspiring, optimistic, convincing, popular, sociable, and trustworthy; Steadiness (The supporter): friendly, kind, good listener, patient, relaxed, enthusiastic, stable, team player, considerate, and steady; Compliance (The corrector): accurate, analytical, cautious, modest, good at discovering facts, high standards, mature, patient, and rigorous. Chameleon:including any three of the above four personality traits.
